# A novel fully human antitumour immunoRNase targeting ErbB2-positive tumours

**DOI:** 10.1038/bjc.2011.146

**Published:** 2011-05-10

**Authors:** M Borriello, P Laccetti, G Terrazzano, G D'Alessio, C De Lorenzo

**Affiliations:** 1 Dipartimento di Biologia Strutturale e Funzionale, Università Federico II, via Cinthia, Napoli 80126, Italy; 2 Dipartimento di Patologia e Biologia Cellulare e Molecolare, Università Federico II, via Pansini, Napoli 80131, Italy; 3 Dipartimento di Chimica, Università della Basilicata, Via N. Sauro, 85, Potenza 85100, Italy

**Keywords:** immunotherapy, ErbB2/HER2, immunoRNase, breast cancer, Trastuzumab

## Abstract

**Background:**

ErbB2 is an attractive target for immunotherapy, as it is a tyrosine kinase receptor overexpressed on tumour cells of different origin, with a key role in the development of malignancy. Trastuzumab, the only humanised anti-ErbB2 antibody currently used in breast cancer with success, can engender cardiotoxicity and a high fraction of patients is resistant to Trastuzumab treatment.

**Methods:**

A novel human immunoRNase, called anti-*Erb*B2 *h*uman *c*ompact *a*nti*b*ody-*RNase* (Erb-hcAb-RNase), made up of the compact anti-ErbB2 antibody *Erb*icin-*h*uman-*c*ompact *A*nti*b*ody (Erb-hcAb) and human pancreatic RNase (HP-RNase), has been designed, expressed in mammalian cell cultures and purified. The immunoRNase was then characterised as an enzymatic protein, and tested for its biological actions *in vitro* and *in vivo* on ErbB2-positive tumour cells.

**Results:**

Erb-hcAb-RNase retains the enzymatic activity of HP-RNase and specifically binds to ErbB2-positive cells with an affinity comparable with that of the parental Erb-hcAb. Moreover, this novel immunoRNase is endowed with an effective and selective antiproliferative action for ErbB2-positive tumour cells both *in vitro* and *in vivo*. Its antitumour activity is more potent than that of the parental Erb-hcAb as the novel immunoconjugate has acquired RNase-based cytotoxicity in addition to the inhibitory growth effects, antibody-dependent and complement-dependent cytotoxicity of Erb-hcAb.

**Conclusion:**

Erb-hcAb-RNase could be a promising candidate for the immunotherapy of ErbB2-positive tumours.

Immunotherapy is a precious strategy to overcome the limits of the conventional anticancer treatments. Indeed, targeting cancer cells via antibodies specific for tumour-associated surface proteins could fulfil the lack of selectivity of radiotherapy and chemotherapy, and is a new interesting biomedical approach as it combines the rational drug design with the progress in understanding cancer biology.

To date, several humanised monoclonal antibodies (MAbs) have achieved FDA approval, and an increasing number is undergoing clinical evaluation (Harris, 2004; Adams and Weiner, 2005; Venkiteshwaran, 2009; Li and Zhu, 2010). A successful example of an approved humanised antibody is represented by Trastuzumab, the only humanised antibody widely used against ErbB2-positive carcinomas for immunotherapy (Stebbing *et al*, 2000).

ErbB2 is an attractive target for immunotherapy, as it is a transmembrane tyrosine kinase receptor, overexpressed on tumour cells of different origin, with a key role in the development of malignancy (Slamon *et al*, 1987). Trastuzumab is currently used with success for breast cancer therapy; however, it can engender cardiotoxicity and a high fraction of breast cancer patients is resistant to Trastuzumab treatment (Seidman *et al*, 2002; Nahta *et al*, 2006).

Furthermore, also carcinomas with a high expression of ErbB2, such as non-small cell lung carcinoma, gastric and prostatic tumours, have been found to be resistant or much less sensitive to Trastuzumab treatment (Agus *et al*, 1999; Gong *et al*, 2004). When Trastuzumab was used in combination with chemotherapy, some benefits have been shown in clinical trials for patients, such as those with ErbB2-positive advanced gastric cancer (Jørgensen, 2010), but cardiac dysfunction has also been observed more frequently (Seidman *et al*, 2002).

A significant addition to the anticancer arsenal has been the construction of a new anti-ErbB2 immunoagent derived from a human, *per se* cytotoxic single-chain antibody fragment (scFv) named Erbicin (De Lorenzo *et al*, 2002b), and a human Fc domain from a human IgG1. This led to a fully human antitumour antibody, designed to be a reduced version of an IgG, with the antiproliferative effect of the scFv moiety on tumour target cells, combined with the ability of the Fc moiety to induce both antibody-dependent cellular cytotoxicity (ADCC) and complement-dependent cytotoxicity (CDC).

The engineered antibody has been called *Erb*icin-*h*uman-*c*ompact *A*nti*b*ody (Erb-hcAb) for its ‘compact’ size (100 kDa), compared with the full size (155 kDa) of a natural IgG. The smaller size should promote an increased extravascular diffusion and tumour penetration.

It has been reported that Erb-hcAb is capable of selective binding to malignant ErbB2-positive cells and of inhibiting their growth *in vitro* and *in vivo*, with no effects on ErbB2-negative cells. Moreover, Erb-hcAb is endowed with both ADCC and CDC cytotoxic effects, whereas Trastuzumab lacks the ability of inducing CDC (De Lorenzo *et al*, 2004b).

More recently, it has been shown that Erb-hcAb does not display the cardiotoxic effects of Trastuzumab *in vitro* on rat cardiomyocytes and *in vivo* on a mouse model (Riccio *et al*, 2009), whereas Trastuzumab was found to be strongly toxic. This difference was found to be due to the different mechanism of action of the two antibodies: Trastuzumab, at difference with Erb-hcAb, induces apoptosis in cardiac cells (Riccio *et al*, 2009). Finally, Erb-hcAb is active *in vitro* and *in vivo* against some Trastuzumab-resistant, ErbB2-positive breast cancer cell lines (Gelardi *et al*, 2010).

Targeted therapy can be accomplished also by using MAbs equipped with radionuclides or toxins (Pastan and FitzGerald, 1991; Carter, 2001). Immunotoxins (ITs) are anticancer agents made up of a recombinant antibody or an antibody fragment directed towards a unique cell surface protein and a potent bacterial toxin capable of inducing the death of target cells (Reiter and Pastan, 1998; Zhang *et al*, 2007).

However, problems have been encountered in clinical trials, especially for the toxicity and immunogenicity of the bacterial or plant toxins used for the ITs (Weiner *et al*, 1989; Schindler *et al*, 2001).

As an alternative to ITs, ImmunoRNases (IRs) have been proposed as more immunocompatible immunoagents. These are fusion proteins in which the toxin has been replaced by a ribonuclease. Mammalian RNases are expected to be not immunogenic and not systemically toxic, as they are *pro-*toxins, which become toxic only upon their internalisation in target cells mediated by the antibody moiety (Rybak and Newton, 1999; De Lorenzo *et al*, 2002a; De Lorenzo and D’Alessio, 2008).

A fully human immunoRNase, *Erb*icin-*h*uman-*RNase* (Erb-hRNase), was constructed through the fusion of Erbicin with human pancreatic RNase (HP-RNase or RNase 1) (De Lorenzo *et al*, 2004a). The chimeric protein was found to retain the high-binding affinity to ErbB2-positive cells of Erbicin and the enzymatic activity of native HP-RNase. When tested *in vitro* on a series of malignant cells, Erb-hRNase was found to discriminate between target and non-target cells, and to specifically inhibit the proliferation of ErbB2-positive cells, with a stronger cytotoxicity on cells with a higher level of ErbB2. Its antitumour activity has been also demonstrated *in vivo* on mice implanted with ErbB2-positive tumours (De Lorenzo *et al*, 2004a; De Lorenzo and D’Alessio, 2009).

The antitumour action of Erb-hRNase is dependent on the ability of this molecule to reach the cytosol and degrade RNA, but it is somewhat limited by the finding (De Lorenzo *et al*, 2007) that the enzymatic activity of Erb-hRNase is inhibited by the RNase inhibitor (RI).

In order to obtain a novel product, which is expected to be superior to the immunoagents currently used in the therapy of breast cancer, a novel immunoRNase has been designed, in which an Erbicin-based scFv-Fc (Erb-hcAb, see above) replaces the scFv of Erb-hRNase. The latter should have the following advantages: the ability of inducing both ADCC and CDC, in addition to the RNase-based cytotoxicity; a prolonged half-life, due to its higher molecular size and the presence of the Fc and an increased avidity due to the presence of two scFv moieties. Furthermore, this novel immunoagent could be resistant to the action of RI, due to the steric hindrance generated by the presence of the larger antibody moiety.

Here, we report on the construction and characterisation of such novel fully human immunoRNase, made up of Erb-hcAb and HP-RNase, expressed in mammalian cell cultures.

This new immunoconjugate, called anti-*Erb*B2 *h*uman *c*ompact *a*nti*b*ody-*RNase* (Erb-hcAb-RNase), has shown to fully retain the binding ability, ADCC and CDC properties of Erb-hcAb and to acquire the RNase activity of its enzymatic moiety, thus inhibiting tumour cell proliferation *in vitro* and *in vivo* more efficiently than the parental Erb-hcAb.

## Materials And Methods

### Cell cultures and antibodies

The SKBR3 cell line from human breast cancer and the A431 cell line from human epidermoid carcinoma were cultured in RPMI 1640 (Gibco BRL, Life Technologies, Paisley, UK). The TUBO cell line from a BALB-neu T mouse-derived mammary lobular carcinoma (kindly provided by Dr G Forni, University of Turin, Italy) was grown in DMEM (Gibco BRL). The media were supplemented with 10% fetal bovine serum (20% for TUBO cells), 50 U ml^−1^ penicillin and 50 *μ*g ml^−1^ streptomycin (all from Gibco BRL).

The antibodies used were Trastuzumab (Genentech, South San Francisco, CA, USA); affinity-isolated IgGs from a rabbit anti-HP-RNase antiserum (from Igtech, Salerno, Italy); horseradish peroxidase (HRP)-conjugated goat anti-rabbit immunoglobulins (Pierce, Rockford, IL, USA); HRP-conjugated goat anti-human affinity-isolated IgGs (Fc specific) (Sigma, St Louis, MO, USA); Erb-hcAb was produced by PER.C6 cells (Crucell NV, Leiden, Netherlands) transfected with the recombinant vector (De Lorenzo *et al*, 2004b), and purified as previously described (De Lorenzo *et al*, 2004b).

### Peripheral blood lymphocytes

Peripheral blood lymphocytes (PBL) were obtained from peripheral blood mononuclear cells isolated by centrifugation on Lymphoprep gradients (Axis Shield PoC AS, Oslo, Norway) from normal donor buffy coats obtained from the Blood Bank of the Medical School of the University of Naples ‘Federico II’. After the separation, PBL were washed twice and incubated in RPMI 1640 medium (Gibco BRL) for 2 h at 37°C to remove adherent cells. The non-adherent cells were used as natural cytotoxic effectors without any additional treatment.

### Construction and production of the anti-ErbB2 Erb-hcAb-RNase

The cDNA coding for the human compact antibody Erb-hcAb (De Lorenzo *et al*, 2004b) was amplified from plasmid pIg1plus (R & D Systems, Minneapolis, MN, USA) by PCR using as forward and reverse primers oligonucleotides containing at their 5′ and 3′ ends an *Sal*I and an *Not*I site, respectively: 5′-ACGCGTCGACCAGGTGCAGCTGTTG-3′ 5′-ATAAGAATGCGGCCGCTTTACCCGGAGACAGG-3′. The PCR fragment was then digested with *Sal*I and *Not*I (New England Biolabs, Hertfordshire, UK) for cloning into the eukaryotic pCMV-endoplasmic reticulum (ER)-myc expression vector (Invitrogen, Carlsbad, CA, USA) downstream to the leader ER signal peptide sequence.

In order to obtain the chimeric construct, the gene fragment coding for the HP-RNase was cloned downstream to the Erb-hcAb sequence by inserting a spacer encoding an 11-amino-acid peptide linker (AAASGGPEGGS) between the scFv-Fc and the RNase. The cDNA coding for HP-RNase and the peptide spacer, previously generated (De Lorenzo *et al*, 2004a) with NotI restriction sites at its 3′ and 5′ ends, was digested with *Not*I and cloned into the corresponding site of pCMV-ER-myc vector downstream to the sequence encoding the human Erb-hcAb.

The correct directional insertion of the RNase gene in the NotI sites was assessed by digestion with suitable endonucleases (New England Biolabs). Sequence analyses confirmed the expected DNA sequence.

The fusion protein was produced by transfecting 293 T (human embryonic kidney) cells with the recombinant vector. In brief, cells grown in DMEM containing 10% FCS at 70–80% confluency were transfected with 5 *μ*g of expression vector by using the Superfect reagent (Qiagen, Valencia, CA, USA). Stable transfectants were selected in the presence of G418 (Sigma) at a concentration of 1 mg ml^−1^. The expression of the antibody construct was determined in the culture medium by quantitative ELISA. For recombinant protein production, transfected 293T cells were expanded to near confluence in selective DMEM medium containing 0.5 mg ml^–1^ G418, 4 mg ml^–1^ glucose, 10% fetal bovine serum, 50 U ml^−1^ penicillin, 50 *μ*g ml^−1^ streptomycin and were then grown for 3–4 days in serum-free medium.

The recombinant fusion protein, henceforth termed Erb-hcAb-RNase, secreted by transfected 293T cells, was purified from culture medium by affinity chromatography on a protein A-Ceramic Hyper DF column (Pall Corporation, Port Washington, NY, USA) loaded with 300–500 ml of conditioned medium, washed with 10 volumes of 100 mM Tris–HCl, pH 8.0 containing 0.5 M NaCl and 10 volumes of 10 mM Tris–HCl, pH 8.0. The protein eluate was obtained with 50 mM glycine pH 3.0, and immediately neutralised with 1/10 volume of 1 M Tris–HCl, pH 8.0.

### RNase activity and inhibition

RNase activity was tested with the acid-insoluble RNA precipitation assay as described previously (Bartholeyns *et al*, 1977) on yeast RNA (8 mg ml^–1^). RNase zymograms, carried out on SDS–PAGE electropherograms, were performed as previously described (Blank *et al*, 1982; De Lorenzo *et al*, 2004a). For inhibition assays, appropriate amounts of Erb-hcAb-RNase and Erb-hRNase were pre-incubated with increasing concentrations of cRI at 37°C for 10 min before starting the activity test mentioned above. Ribonuclease inhibitor was purchased from Promega (Madison, WI, USA); its concentration was determined as previously described (Naddeo *et al*, 2005).

### ELISA assays

ErbB2-positive SKBR3 cells and ErbB2-negative A431 control cells, harvested in non-enzymatic dissociation solution (Sigma), were washed and transferred to U-bottom microtitre plates (1 × 10^5^ cells per well). After blocking with PBS containing 6% bovine serum albumin (BSA), cells were incubated with conditioned medium or purified immunoagents in ELISA buffer (PBS/BSA 3%) for 90 min. The pelleted cells were washed, resuspended in 100 *μ*l of ELISA buffer and incubated with an anti-human IgG (Fc specific) MAb (Sigma) for detection of Erb-hcAb and Erb-hcAb-RNase. The latter was detected also with an anti-HP-RNase IgG antibody followed by HRP-conjugated goat anti-rabbit immunoglobulin. After 1 h, the plates were centrifuged, washed with ELISA buffer and reacted with 3,3′,5,5′-tetramethylbenzidine (Sigma). Binding values were determined from the absorbance at 450 nm, and reported as the mean of at least three determinations (s.d. ⩽5%).

### Analyses on cell cultures and lysates

Cells were seeded in 96-well, flat-bottom plates; SKBR3 cells at a density of 1.5 × 10^4^ well; A431 at a density of 5 × 10^3^ well. After incubation at 37°C for 72 h with the protein under test, viable cells were counted by the Trypan blue-exclusion test. Cell viability was determined in triplicate by using methyl tetrazolium test (Sigma) according to the manufacturer's recommendations. The resulting absorbance at 570 nm was measured in a microplate counter (Multilabel Counter Victor 3, Perkin-Elmer, Waltham, MA, USA). Cell survival was expressed as percentage of viable cells in the presence of the protein under test, with respect to control cultures grown in the absence of the protein. Typically, s.d. were below 5%.

SKBR3 cell extracts were prepared as previously described (De Lorenzo *et al*, 2007). Protein concentration was determined by the Bradford colorimetric assay (Sigma), and aliquots of 20 *μ*g were run on 12% SDS–PAGE, followed by electroblotting onto PVDF membranes (Millipore Corporation, Bedford, MA, USA).

Intracellular Erb-hcAb-RNase was detected with anti-HP-RNase or anti-Fc IgGs, respectively, followed by goat anti-rabbit HRP-conjugated IgGs. The signal from secondary antibodies was visualised by enhanced chemiluminescence detection (ECL western blotting detection kit, Amersham Biosciences, Uppsala, Sweden). The signal intensity of reactive bands was measured with a phosphorimager (GS-710, Bio-Rad, Hercules, CA, USA).

### ADCC and CDC tests

Target and control cells were detached from culture dishes with a cell dissociation solution (Sigma) and transferred to round-bottom 96-well plates (2 × 10^4^ cells per well). For ADCC assays, target or control cells were treated with the immunoagents (5 *μ*g ml^−1^ of serum-free medium) and PBL at 37°C for 3–4 h. For CDC assays, cells were incubated at 37°C with human serum. Cultures were performed in triplicate in a final volume of 200 *μ*l. Controls included target cells incubated in the absence of effector cells or in the presence of either serum or immunoagent alone. Tumour cell lysis was determined by measuring the release of lactate dehydrogenase (LDH) using an LDH detection kit (Roche, Mannheim, Germany). Antibody-dependent cellular cytotoxicity or CDC was calculated as the per cent of cytolysis measured in the presence of immunoagent and PBL or human serum, for ADCC and CDC, respectively, taking as 100% the maximal LDH release determined by lysis of target cells with 1% Triton X-100. Standard deviations were calculated on the basis of the results obtained from three different experiments.

### *In vivo* antitumour activity

All experiments were performed with 6-week-old female Balb/cAnNCrlBR mice (Charles River Laboratories, Calco, Italy). The TUBO cells (5 × 10^5^) were suspended in 0.2 ml sterile PBS and injected subcutaneously (day 0) in the right paw. At day 7, when tumours started to appear, the mice were divided into three groups. At day 15, when tumours were clearly detectable, Erb-hcAb-RNase dissolved in PBS was administered i.p. at doses of 1.8 mg kg^−1^ of body weight for seven times at 72 h intervals. The second group of animals was treated with equimolar doses (1.3 mg kg^−1^ of body weight) of Erb-hcAb, dissolved in PBS and administered i.p. for seven times at 72 h intervals. The third group of control animals was treated with identical volumes of sterile PBS.

To test the effects of Trastuzumab, used as a control, the experiment was repeated on the same model. The TUBO cells (5 × 10^5^) were suspended in 0.2 ml sterile PBS and injected subcutaneously (day 0) in the right paw. When tumours were clearly detectable, Trastuzumab dissolved in PBS was administered i.p. at doses of 2 mg kg^–1^ for seven times at 72 h intervals. The second group of control animals was treated with identical volumes of sterile PBS.

During the period of treatment, tumour volumes (*V*) were measured with caliper and calculated by the formula of rotational ellipsoid *V*=*A* × *B*^2^/2 (*A* is the axial diameter, *B* the rotational diameter). All mice were maintained at the animal facility of the Department of Cellular and Molecular Biology and Pathology, University of Naples ‘Federico II’. Animal studies were conducted in accordance with the Italian regulation for experimentation on animals. All *in vivo* experiments were carried out with ethical committee approval and met the standards required by the UKCCCR guidelines (Workman *et al*, 1998).

### Statistical analysis

Analysis of variance followed by a *post hoc* Dunnett's *t*-test was used to analyse the data. A *P*-value <0.05 was considered to be significant.

## Results

### Production and purification of a novel fully human anti-ErbB2 compact antibody-RNase conjugate

**Figure 1:**
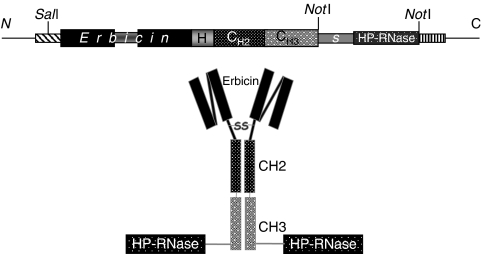
Schematic representation of the chimeric ImmunoRNase, Erb-hcAb-RNase, obtained by fusing the human compact antibody Erb-hcAb and the HP-RNase. Erbicin=the human anti-ErbB2 scFv; H=hinge; CH2-CH3=the heavy constant domains of the human IgG1 Fc; S=the spacer peptide AAASGGPEGGS linking the scFv-Fc and the RNase moieties; HP-RNase=the RNase moiety.

A new human anti-ErbB2 immunoagent was generated by fusing HP-RNase with the fully human anti-ErbB2 compact antibody (Erb-hcAb) (De Lorenzo *et al*, 2004b). The cDNAs coding for the human compact antibody Erb-hcAb and HP-RNase were amplified by PCR and cloned into the eukaryotic pCMV-ER-myc expression vector. In particular, the cDNA encoding HP-RNase was cloned downstream to the sequence encoding the carboxy terminus of the scFv-Fc (Erb-hcAb) by adding a spacer encoding a 11-amino-acid residue peptide linker (*AAASGGPEGGS*) to minimise the steric hindrance between the two moieties of the chimeric protein (Figure 1). The recombinant plasmid, sequenced to confirm faithful cloning, was stably transfected in 293 T (human embryonic kidney) cells, and the recombinant construct was expressed as a secretion product into the culture medium. Once selected by quantitative ELISA assays, the clone producing the highest levels of Erb-hcAb-RNase was used for the production of the chimeric immunoagent, which was then purified by affinity chromatography on a protein A-Ceramic Hyper DF column. The immunoagent was named Erb-hcAb-RNase .

### Characterisation of Erb-hcAb-RNase

**Figure 2:**
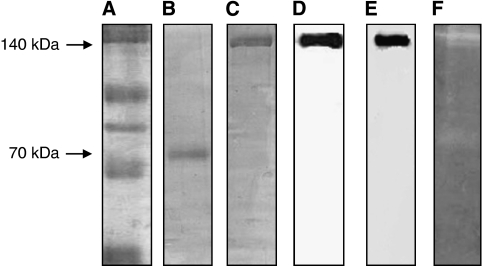
SDS–PAGE and western blotting analyses of purified Erb-hcAb-RNase. Erb-hcAb-RNase was run under reducing (lane B) or non-reducing (lane C) conditions; molecular weight standards are in lane A; western blotting analyses of the purified sample with an anti-human IgG1 (Fc specific) (lane D) or with the anti-HP-RNase antibody (lane E); Zymogram of Erb-hcAb-RNase using yeast RNA as a substrate in lane F.

When Erb-hcAb-RNase was analysed by SDS–PAGE (Figure 2), it was found to migrate under reducing conditions with the expected molecular size of about 70 kDa (Figure 2, lane B), and as a dimer of about 140 kDa under non-reducing conditions (Figure 2, lane C). This result indicates that the fusion protein is expressed as a disulphide-linked dimer. Western blotting analyses performed with either an anti-human Fc or an anti-HP-RNase antibody demonstrated immunoreactivity of the purified, dimeric protein with a molecular size of 140 kDa (Figure 2, lanes D and E).

The fusion protein was then tested for enzymatic activity by a zymogram, developed using yeast RNA as a substrate. As illustrated in Figure 2 lane F, a single active band was detectable, corresponding to the size of Erb-hcAb-RNase. In a parallel assay, the first generation anti-ErbB2 immunoRNase (Erb-hRNase), made up of the Erbicin scFv and HP-RNase, was used as a positive control (data not shown).

The ribonucleolytic activity of the purified Erb-hcAb-RNase was further tested with the acid-insoluble RNA precipitation assay (Bartholeyns *et al*, 1977), by which the chimeric immunoagent was found to have a specific activity of 730±25 units nmol^–1^. This result was confirmed for several preparations of the recombinant fusion protein. Because the specific activity of hErb-hRNase, that is the monomeric anti-ErbB2 immunoRNase, previously tested, is 950±25 units nmol^–1^ (De Lorenzo *et al*, 2004a), we can conclude that Erb-hcAb-RNase retains about 80% of the activity of the first generation ImmunoRNase.

To determine the sensitivity of Erb-hcAb-RNase to RI inhibition, the enzymatic assays were repeated in the absence or in the presence of increasing concentrations of RI. Erb-hRNase was used as a positive control, as it was previously found to be fully inihibited by RI (De Lorenzo *et al*, 2007).

**Figure 3:**
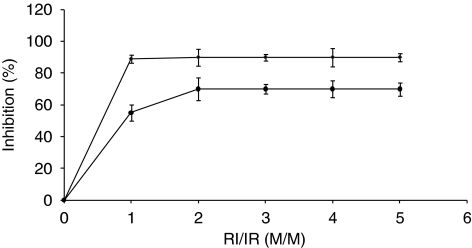
Effects of the RNase inhibitor (RI) on the enzymatic activity of the immunoRNases (IR). Inhibition by RI of the catalytic activity of the anti-ErbB2 Erb-hcAb-RNase (circles) or Erb-hRNase (rhomboids) was measured at increasing RI/IR ratios.

As shown in Figure 3, the novel chimeric protein was found to be susceptible of inhibition by RI, even though it was found to be less sensitive than the monomeric immunoRNase. Indeed, the new ImmunoRNase retains a residual 30% of the enzymatic activity even at a cRI/IR ratio of about 5, whereas the activity of the monomeric IR is completely inhibited at a cRI/IR ratio of about 1 (Figure 3).

**Figure 4:**
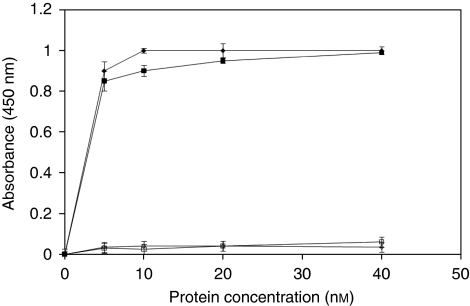
Binding curves of Erb-hcAb-RNase or Erb-hcAb to ErbB2-positive (SKBR3) and -negative (A431) cell lines. SKBR3 cells were tested by enzyme-linked immunoadsorbent assay with Erb-hcAb-RNase (black squares), or with Erb-hcAb (black rhomboids), used as a control; A431 cells were tested as indicated above with Erb-hcAb-RNase or Erb-hcAb (empty squares and rhomboids, respectively).

This result could be considered not surprising as the larger molecular size of the antibody fragment of Erb-hcAb-RNase with respect to that of the scFv in Erb-hRNase could hinder the interactions between RI and the RNase. When the ability of the recombinant fusion protein to bind to ErbB2-positive cells was analysed by ELISA assays (Figure 4), Erb-hcAb-RNase was found to fully retain the specificity and the affinity of the parental compact antibody for mammary carcinoma ErbB2-overexpressing SKBR3 cells. As a negative control, we used A431 cells (from human epidermoid carcinoma), which express very low levels of ErbB2. The apparent binding affinity of Erb-hcAb-RNase for the ErbB2 receptor, that is the concentration corresponding to half-maximal saturation, was about 1 nM, identical to that of the parental Erb-hcAb.

These results demonstrate that the compact antibody and the RNase retain their biological functions in the chimeric protein.

### Biological effects of Erb-hcAb-RNase on ErbB2-positive tumour cells

**Figure 5:**
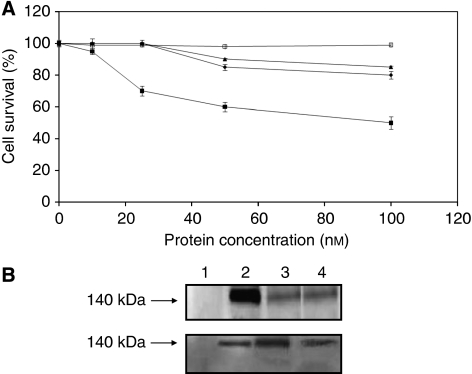
*In vitro* effects of Erb-hcAb-RNase on tumour cells. (**A**) Dose–response curves of ErbB2-positive SKBR3 (black symbols) and ErbB2-negative A431 cells (empty symbols), treated for 72 h with Erb-hcAb-RNase (squares). The effects of Erb-hcAb (rhomboids) and Trastuzumab (triangles), used as a positive control, are also shown. (**B**) Intracellular levels of Erb-hcAb-RNase. The internalisation of Erb-hcAb-RNase by target cells was measured (three experiments) by western blotting with an anti-RNase (upper panel) and an anti-Fc antibody (lower panel). Lanes 1–4 (both panels): immunoreactive proteins in the cytosolic fraction of cells untreated (lane 1) or treated with Erb-hcAb-RNase (for: 16 h, lane 2; 24 h, lane 3; 48 h, lane 4).

To assess the *in vitro* effects of Erb-hcAb-RNase on tumour cell growth, the ErbB2-positive SKBR3 and the ErbB2-negative A431 cell lines were incubated with increasing concentrations of Erb-hcAb-RNase, Erb-hcAb or Trastuzumab, used as a control. As shown in Figure 5A, Erb-hcAb-RNase inhibited the growth of SKBR3 cells in a dose-dependent manner, showing an antiproliferative effect more potent than that observed for either the parental Erb-hcAb or Herceptin. The immunoagent did not have any effect on the proliferation of ErbB2-negative A431 cells (see Figure 5A).

These findings suggest that the increased cyotoxicity of Erb-hcAb-RNase with respect to that of Erb-hcAb is due to its RNase moiety, which can exert its enzymatic activity upon internalisation mediated by the antibody moiety.

To test the ability of the immunoRNase to be internalised by ErbB2-positive cells, we analysed the level of Erb-hcAb-RNase in the cytosol of treated cells. Briefly, SKBR3 cells were treated with the immunoRNase (100 nM) for 16–48 h at 37°C, stripped of surface-bound protein with a low pH glycine/NaCl buffer and lysed.

Equal protein amounts of cell extracts were analysed by immunoblotting using either anti-Fc or anti-HP-RNase IgGs, followed by HRP-conjugated secondary antibodies. A strong immunoreactive band with the molecular weight expected for the immunoRNase was observed in the intracellular fraction of treated cells (see Figure 5B), whereas no signal was detected in the extracts of untreated control cells.

These results indicate that the immunoRNase is internalised by ErbB2-positive cells and reaches the cytosol, where potential RNA targets are, so that the RNase can express its cytotoxic action.

**Figure 6:**
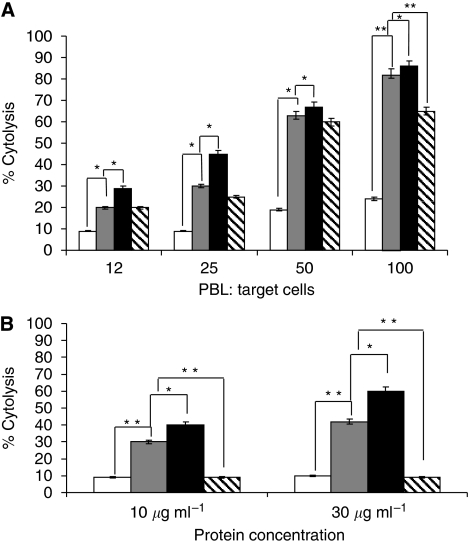
Antibody-dependent and CDC assays of Erb-hcAb-RNase. (**A**) SKBR3 cells treated with PBL as effector cells at four different ratios in the presence of Erb-hcAb-RNase (grey bars), Erb-hcAb (black bars), Trastuzumab (striped bars) used as positive controls or Erbicin, the parental anti-ErbB2 scFv (white bars), used as a negative control. Histogram represents a summary data of multiple experiments. Bars represent means±s.d. (^*^P<0.05; ^**^P<0.01). (**B**) SKBR3 cells were incubated for 6 h in the presence of human serum as a source of complement with Erb-hcAb-RNase (grey bars), Erb-hcAb (black bars), used as a positive control, Herceptin (striped bars) or Erbicin (white bars), used as a negative control, at concentrations of 10 and 30 *μ*g ml^−1^. Data are reported as the mean of three independent experiments. Bars represent means±s.d. (^*^P<0.05; ^**^P<0.01).

To investigate whether Erb-hcAb-RNase was capable of recruiting immune effector functions *in vitro*, assays for cytolysis of tumour cells as induced by PBL, or complement, were performed. To determine the capacity of Erb-hcAb-RNase to selectively trigger ADCC towards antigen-expressing cells, SKBR3 and A431 cells were incubated for 3 h with increasing amounts of effector PBL in the absence or in the presence of Erb-hcAb-RNase (5 *μ*g ml^−1^). We used as positive controls both Erb-hcAb and Trastuzumab. As shown in Figure 6A, Erb-hcAb-RNase lysed SKBR3 target cells in the presence of PBL with an efficacy slightly lower than that of the parental Erb-hcAb. The extent of lysis reached almost 80% of treated cells at a ratio of 100 : 1 (effector to target cells), whereas Trastuzumab induced about 65% lysis at the same ratio. No effects were detected when Erb-hcAb was replaced by the parental anti-ErbB2 scFv, lacking the Fc domain or when Erb-hcAb-RNase was tested in parallel assays carried out with ErbB2-negative A431 cells (data not shown), thus demonstrating the specificity of the Erb-hcAb-RNase-dependent cell-mediated cytolytic activity (see Figure 5).

Thus, we can conclude that the presence of the RNase in the new construct does not affect significantly the interactions between the Fc region of Erb-hcAb and the CD16 receptor of the natural killer cells.

To test the ability of Erb-hcAb-RNase of inducing CDC against ErbB2-positive tumour cells, SKBR3 target cells were incubated for 6 h with Erb-hcAb-RNase (10 or 30 *μ*g ml^−1^) in the absence or in the presence of human serum as a source of complement. As illustrated in Figure 6B, Erb-hcAb-RNase was found to effectively lyse SKBR3 cells in the presence of serum with an average specific lysis of 40% after 6 h, whereas the parental Erb-hcAb induced about 60% lysis. Complement-dependent cytotoxicity was not detected when the parental anti-ErbB2 scFv, lacking the Fc domain, was used as a negative control (see Figure 6B), or when ErbB2-negative A431 cells were incubated with Erb-hcAb-RNase and human serum (data not shown). Similarly, as previously reported (Drebrin *et al*, 1988; De Lorenzo *et al*, 2004b), no lysis was detectable when SKBR3 cells were treated with Trastuzumab in the presence of human serum (see Figure 6B).

### *In vivo* antitumour effects of Erb-hcAb-RNase

For *in vivo* studies, Erb-hcAb-RNase was tested on murine TUBO tumour cells expressing ErbB2 of rat origin (Rovero *et al*, 2000). When administered to female mice, TUBO cells induce tumours very similar to the alveolar-type human lobular mammary carcinomas (Di Carlo *et al*, 1999).

**Figure 7:**
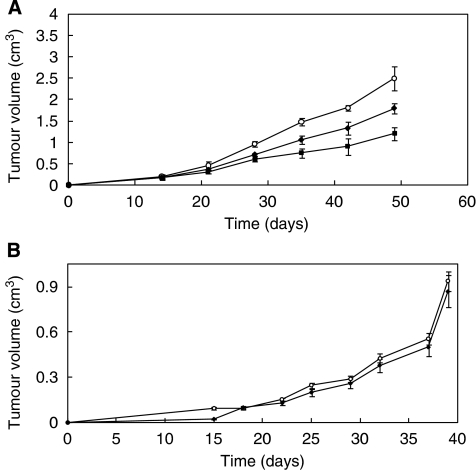
*In vivo* antitumour effects of Erb-hcAb-RNase and Trastuzumab. (**A**) *In vivo* antitumour effects of Erb-hcAb-RNase. Tumour growth was followed in mice inoculated s.c. with 5 × 10^5^ TUBO mammary carcinoma cells. Control animals (empty circles) were treated with sterile PBS solution. Treated animals were injected with Erb-hcAb-RNase (black squares), starting at day 14. Seven doses, each of 1.8 mg kg^−1^ of body weight, were administered at 72 h intervals i.p. In a parallel experiment, Erb-hcAb (black rhomboids) was administered at equimolar doses (1.3 mg kg^−1^) as indicated for Erb-hcAb-RNase, for comparison. (**B**) *In vivo* antitumour effects of Trastuzumab. Tumour growth was followed in mice inoculated s.c. with 5 × 10^5^ TUBO mammary carcinoma cells. Control animals (empty circles) were treated with sterile PBS solution. Treated animals (black rhomboides) were injected with Trastuzumab, starting at day 14. Seven doses, each of 2 mg kg^−1^ of body weight, were administered at 72 h intervals i.p.

In order to compare the *in vivo* antitumour efficacy of this novel immunoagent with that of the parental Erb-hcAb, the effects of equimolar doses of Erb-hcAb and Erb-hcAb-RNase were tested in parallel on the same experimental model. In particular, to detect the differential potency of the two immunoagents, they were administered at lower doses than those used for peritumoural administrations of Erb-hcAb in the previous experiment (De Lorenzo *et al*, 2004b) when a dramatic reduction (96%) in tumour volume was observed. As shown in Figure 7, the treatment of mice bearing TUBO tumours with seven doses, at 72 h intervals, of 1.8 mg kg^−1^ of Erb-hcAb-RNase induced a significant reduction (about 50%) in tumour volume and showed more potent antitumour effects than those observed for the parental Erb-hcAb (doses of 1.3 mg kg^−1^).

As a further control, we tested in a parallel experiment the effects of equimolar doses of Trastuzumab on the same experimental model. As shown in Figure 7B, Trastuzumab was found to have a very limited inhibitory action on the growing tumours with respect to that of Erb-hcAb-RNase. This may be interpreted by considering that humanised Trastuzumab maintains at the antigen-binding site structural determinants of mouse origin, which engender a poor fit with the rat antigen, a negative feature, which is not shared by the human antigen-binding structure of Erb-hcAb.

During the period of treatment, the animals did not show signs of wasting or other visible signs of toxicity.

## Discussion

Immunotoxins, based on toxins fused to antibody moieties specifically reactive to a certain type of tumour cells, have been designed for a novel approach in anticancer therapy. However, the non-specific toxicity of ITs associated to vascular leak syndrome and/or hepatotoxicity, as well as the immunogenicity of their bacterial or plant toxins, have often limited the outcome of ITs as anticancer drugs (Weiner *et al*, 1989; Reiter and Pastan, 1998; Schindler *et al*, 2001).

To circumvent these problems, IRs, based on the IT principle, have been proposed (Rybak and Newton, 1999; De Lorenzo *et al*, 2002a; De Lorenzo and D’Alessio, 2008). In the IRs, the toxin moiety of ITs is replaced by a non-toxic and non-immunogenic RNase, which becomes toxic only upon its internalisation mediated by the antibody moiety in the target cells.

We previously reported on a fully human immunoRNase obtained by fusing a human scFv and a human RNase (De Lorenzo *et al*, 2004a), named Erb-hRNase, which is directed to the ErbB2 receptor, overexpressed in many carcinomas, especially in breast carcinoma (Slamon *et al*, 1987; Nahta *et al*, 2006).

Erb-hRNase was found to exert a powerful and selective antitumour action *in vitro* and *in vivo*. However, its successful therapeutic use could be somewhat limited by the lack of antibody effector functions, as well as by its monovalent nature and by its small size (<50 kDa), which could be responsible for a reduced tumour retention and a faster clearance from the blood circulation, respectively.

In this study, we report on the production and characterisation of a novel second generation fully human IR targeting the ErbB2 receptor called Erb-hcAb-RNase.

It results from the fusion of the fully human anti-ErbB2 compact antibody Erb-hcAb (De Lorenzo *et al*, 2004b) with HP-RNase. We have shown here that both the antibody and RNase moieties preserve their biological actions in the immunoconjugate. This second generation immunoRNase is endowed with the following properties:

1. it recognises one of the most specific tumour-associated antigens, such as ErbB2, with an affinity comparable with that of the parental compact antibody;
2. it retains the enzymatic activity of the first generation immunoRNase (Erb-hRNase) but, at difference with the monovalent IR, it is only partially susceptible to RI inhibition;
3. it displays effective antibody effector functions with an efficacy comparable with that of the parental Erb-hcAb;
4. it inhibits efficiently the proliferation of ErbB2-positive tumour cells both *in vitro* and *in vivo* with antitumour effects more potent than those observed for the parental compact antibody. The latter results can be explained by the additional toxic action of the internalised RNase;
5. the size of Erb-hcAb-RNase should be better suited for therapeutic applications due to the potential prolonged half-life with respect to the first generation scFv-based IR and to better penetration properties than full size IgG-toxin immunoconjugates.

It has been already reported by Menzel *et al* (2008) on a powerful anti-CD30 scFv-Fc-RNase, made up of a CD30 lymphoma specific human scFv-Fc fused to HP-RNase, showing specific binding and *in vitro* cytotoxicity on CD30+ lymphoma cells at nM concentrations. However, this IR was not tested for its effector functions (ADCC and CDC) and for its *in vivo* antitumour activity.

To our knowledge, Erb-hcAb-RNase is to date the first fully human antibody-RNase to be constructed and tested with satisfactory results *in vivo* demonstrating for the first time that the presence of the RNase does not hinder the antibody effector functions (ADCC and CDC).

On the basis of its fully human nature and selectivity of its antitumour action on target cells, Erb-hcAb-RNase represents a promising valuable tool in cancer therapy, thus supporting the hypothesis that the scFv-Fc-RNase format is the most appropriate for the production of a novel generation of IR better suited for therapeutic applications, as it combines the advantages of the first generation IR with those of functional relevant antibody domains. However, the efficacy of this immunoRNase could be further improved for a more efficient killing, as required for a successful cancer therapy, by replacing the native human RNase with a more cytotoxic variant resistant to the RI.
